# miR-381 Inhibits Proliferation and Invasion of Non-Small-Cell Cancer Cells by Targeting USP39

**DOI:** 10.1155/2022/2195393

**Published:** 2022-08-21

**Authors:** Fang Cui, Peng Luo, Rui Wu, Jiangping Meng

**Affiliations:** ^1^Department of Laboratory Medicine, The First Affiliated Hospital of Chongqing Medical University, No. 1 Youyi Road, Yuzhong District, Chongqing 400016, China; ^2^Assisted Reproductive Center, Department of Obstetrics and Gynecology, The First Affiliated Hospital of Chongqing Medical University, Chongqing 400016, China

## Abstract

It is known that miR-381 plays a therapeutic role in a variety of cancers, but the regulatory mechanism of miR-381 in the treatment of lung cancer remains unclear. This study is aimed at exploring the expression level and mechanism of miR-381 in lung cancer. In this experiment, quantitative real-time PCR (qRT-PCR), western blot, and other methods were used to detect the expression of miR-381 and ubiquitin-specific protease 39 (USP39) in lung cancer tissues. The target genes of miR-381 were predicted by bioinformatics techniques, and the targeting relationship between miR-381 and USP39 was verified by the dual-luciferase reporting method. The expression levels of miR-381 and USP39 were adjusted to verify the effect of miR-381 on the expression of USP39 gene. The effect of miR-381 expression on proliferation of lung cancer cells was verified by cell proliferation and invasion experiments. miR-381 was downregulated in non-small-cell lung cancer tissues and cell lines, while USP39 was upregulated. The dual-luciferase reporter gene assay showed that miR-381 and USP39 had targeted binding sites. After transfection with miR-381 mimics, USP39 expression was significantly decreased, cell proliferation decreased, and apoptosis increased. After transfection with miR-381 inhibitor, USP39 expression was significantly increased, cell proliferation increased, and cell apoptosis decreased. Overexpression of USP39 significantly increased the invasion ability and cell survival curve (*p* < 0.05). In conclusion, overexpression of miR-381 can regulate the expression of USP39, inhibit the proliferation and invasion of cancer cells, and induce apoptosis of cancer cells. This may provide a new perspective and strategy for targeted therapy of non-small-cell lung cancer.

## 1. Introduction

At present, lung cancer is one of the malignant tumors with the highest morbidity and mortality in the world. Its pathological types can be divided into two kinds: one is small-cell lung cancer (SCLC); the second type of non-small-cell lung cancer (NSCLC) includes adenocarcinoma and squamous cell carcinoma, and adenocarcinoma is one of the most common cancers in lung cancer. For patients with advanced lung cancer [1], the main treatment means include chemical drug therapy, radiation therapy, and biological targeted therapy. Chemotherapy is the main treatment method, but even with the intervention of multiple treatment methods, the survival rate of patients after treatment is only 11% [2, 3], and it is very easy to relapse and metastasize. As a result, the prognosis of lung cancer patients is generally not ideal, and the cure rate is not high [4]. Therefore, it is very important to actively study and explore new pathogenic factors and mechanisms of NSCLC for innovative drug use and treatment of lung cancer. We performed high-throughput sequencing on lung cancer tissues and found that there were some differentially expressed genes, among which miR-381 was one of the differentially expressed genes. Meanwhile, we found that USP39 was also differentially expressed. Colleagues in the research group chose to study other differential genes, and I chose to study miR-381 and USP39. TargetScan analysis found that miR-381 has specific binding sites with USP39, so it was speculated that USP39 may be a target of miR-381, and USP39 expression can be targeted and regulated by miR-381 to control the development of lung cancer cells.

Ubiquitin-specific protease 39 (USP39) belongs to the deubiquitination enzyme family [5]. It has previously been reported that USP39 regulates cell division and mRNA splicing [6–8]). Therefore, for tumor cells, the development of tumor cells can be inhibited by regulating their growth and reproduction. Many studies have shown that USP39 is overexpressed in a variety of cancer cells and acts as a carcinogenic factor. For example, USP39 can promote the proliferation and growth of cancer cells in colon cancer cells, pancreatic cancer cells, and gastric cancer cells [9–11]. USP39 has been less studied in lung cancer, but there are studies showing that USP39 was upregulated in lung adenocarcinoma cells and promoted the growth of lung adenocarcinoma cells [12]. Knockout of USP39 can inhibit tumor genesis, induce cell apoptosis, and inhibit lung adenocarcinoma cell metastasis [13]. Therefore, USP39 may serve as a new target for lung cancer treatment.

MicroRNAs (miRNAs) are important gene regulators in cells. mRNA can promote mRNA degradation or inhibit mRNA translation by binding to the 3′ untranslated region (3′ UTR) of mRNA, thus playing an important role in tumor development. Many miRNAs have been reported to be involved in the diagnosis, treatment, and prognosis of cancer. For example, miR-375 is low expressed in lung squamous cell carcinoma tissues ([14]) and inhibits invasion, migration, and proliferation of NSCLC through the CIP2A pathway, and miR-30a-5p is low expressed in NSCLC ([15]) and inhibits proliferation and invasion of cancer cells. As a miRNA closely related to tumor development, miR-381 has been shown to play an important therapeutic role in breast cancer, esophageal squamous cell carcinoma, gastric cancer, and other cancers by regulating the proliferation and growth of tumor cells [16–18]. However, the therapeutic effect of miR-381 on lung cancer has not been confirmed. In this study, the expression and role of miR-381 and USP39 in lung cancer were detected to explore whether miR-381 and USP39 can regulate each other and control the reproduction and development of lung cancer cells together.

## 2. Materials and Methods

### 2.1. Cell Cultivation

Human bronchial epithelial cell line (HBE) and human lung adenocarcinoma cell line A549 were all from The First Affiliated Hospital of Chongqing Medical University. All tissue samples were collected with patients' informed consent and reviewed and approved by the Ethics Committee of The First Affiliated Hospital of Chongqing Medical University, and the study was in accordance with the regulations of the Ethics Committee. All cells and tissues were cultured in an incubator at 37°C and 5% CO_2_, with 10% fetal bovine serum, 100 mg/mL streptomycin, and 100 U/mL penicillin added into the medium, and the cells were multiplied to a certain number for reserve use.

### 2.2. Quantitative Real-Time PCR (qRT-PCR)

Total RNA of lung cancer tissues was extracted by TRIzol (Invitrogen™) at 4°C, and reverse transcription was performed using a reverse transcription kit (Takara). The cDNA was used as a template, U6 was used as an internal reference, and miR-381 mimics and miR-381 inhibitor primers (Guangzhou Ribio) were added for PT-PCR (Takara). Every miRNA detection has set 3 duplicate holes to ensure that the Ct differential between the holes was less than 0.5. The relative expression level of miR-381 was detected. The primer sequence is as follows: miR-381: forward 5′-TTAGUACGCCGACUUCCGAGUGCCACCUCTAGGUUGAGCCAAACU-3, reverse 5′-UAGCCACUCGAATGCUUCCAGGCCAU-3′; U6: forward 5′-CGTTCCGTCGCACAGCAG-3′, reverse 5′-CACGCAACTAATGTTGTGTC-3′; USP39: forward 5′-TTGAAGTCTCACGCCTACATTC-3′, reverse 5′-GGCAGTAAAACTTGAGGGTGT-3′; and GAPDH: forward 5′-TGACTTCAACAGCGACACCCA-3′, reverse 5′-CACCCTGTTGCTGTAGCCAAA-3′.

### 2.3. Western Blot Assay

Total protein was extracted from each group of cells, lung cancer tissues, and paracancerous tissues using lysate, and protein quantification was carried out with the BCA kit (Sigma Aldrich). Protein samples were added to the gel and separated by electrophoresis. Cut the PVDF film and put it into the transfer membrane tank together with the separation adhesive for film transfer. Take out the PVDF membrane and add TBS for transfer printing. Add the blocking solution and seal it in the incubator for 1 h, cut the target strip, add the corresponding anti-USP39 antibody (1 : 2000, Abcam), anti-Bax (1 : 2000, Abcam) to each group, and incubate overnight at 4°C. The next day, secondary antibody (1 : 10000, Santa Cruz Biotechnology, US) was added and incubated at room temperature for 1 h. A chemical luminescent agent was added for culture and exposure. Using *β*-actin as an internal reference, the protein bands were displayed using the ECL detection system (Shanghai Jinpeng Analytical Instrument Co., LTD).

### 2.4. Immunohistochemistry Assay (IHC)

The cells and tissues were fixed with a fixator, then embedded with paraffin, and finally sectioned in paraffin blocks. Paraffin sections were cleaned and dewaxed with ethanol solutions of different concentrations. Antigen repair solution was used to repair the antigen, and PBS was washed 3 times. Polyclonal rabbit anti-USP39 antibody (Abcam, US) was added and incubated overnight at 4°C, then washed with PBS solution. HRP-labeled secondary antibody (Abcam, US) was added and incubated at 4°C for 30 min: DAB dyeing with DAB reagent (Solarbio Life Science, Beijing), microscope observation, and photography.

### 2.5. Cell Transfection

A549 cells were inoculated in 96-well cell culture plates. USP39 cDNA was cloned into pcDNA3.1 (Invitrogen) vector. After adding pcDNA/USP39, miR-381 mimics, miR-381 inhibitor, and control into Opti-MEM medium, the cells were transfected with Lipofectamine™ 3000 (ThermoFisher Scientific). Finally, the expression of miR-381 was detected.

### 2.6. Flow Cytometry Was Used to Detect the Number of Early Apoptotic Cells

After transfection, the cells were added with trypsin without EDTA for digestion for a period of time, then washed with PBS buffer and resuspended. 1x binding buffer was added, FITC-Annexin V was added, and the instructions of the apoptosis detection kit (Meilunbio®, China) were followed. Cell apoptosis was detected by the final product.

### 2.7. Luciferase Reporter Assay

HEK-293T cells were cultured in 24-well plates and cotransfected into USP9X-wt and USP9X-mut plasmid vectors with miR-NC and miR-381 mimics using Lipofectamine™ 2000, respectively. Check luciferase activity according to the instructions of the luciferase assay kit (Abcam, China).

### 2.8. MTT Assay

The cultured cells were collected and placed on 96-well plates with approximately 2000 cells per well, and cell transfection was then performed. Add 20 *μ*L MTT (Sigma) solution and 150 mL DMSO (Sigma) to each well after incubation for 24, 48, and 72 h. OD values of cells in each group at 24, 48, and 72 h after transfection were detected by an enzyme plate analyzer.

### 2.9. Transwell Invasion Assay

Before the experiment, a layer of matrix glue was spread on the membrane, cell suspension (without serum) was added to the upper compartment, and bovine serum medium was added to the lower compartment. Gene mimics and control genes were transfected and cultured routinely. Each group was set with 3 replicates, and the reaction time was 24 h and 48 h at 37°C under 5% CO_2_. Remove the Transwell chamber and wash it several times with PBS, then wipe the impermeable cells and excess medium with a cotton swab and stain it with crystal violet. Pick a few fields at random under the microscope. The number of transmembrane cells was counted, and the mean value was taken.

### 2.10. Statistical Analysis

SPSS25.0 was used for data analysis. Data were expressed as the mean ± sd. One-way analysis of variance (ANOVA) was used for quantitative data, and *p* < 0.05 was taken as the significance criterion. The quantitative values of all test results were normalized to 1 as the control group.

## 3. Results

### 3.1. Expression of miR-381 and USP39 in NSCLC Cells

**Figure 1:**
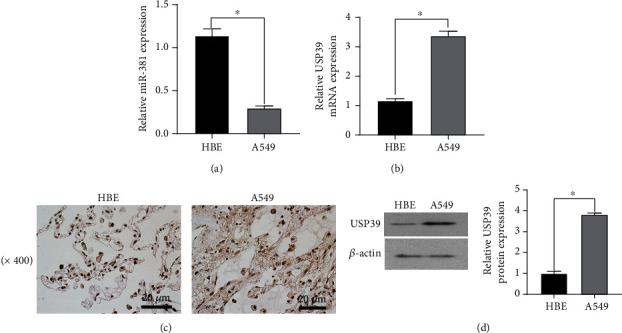
Expression of miR-381 and USP39 in tissues and cell lines of non-small-cell lung cancer. (a) The left figure shows the detection of miR-381 in different lung cancer tissues and adjacent normal tissues by qRT-PCR. The right image shows the expression level of miR-381 in different lung cancer tissues. ^∗^*p* < 0.05 versus normal tissues, versus HBE. (b) Expression of USP39 in lung cancer and adjacent normal tissues. ^∗^*p* < 0.05 versus normal tissues. (c) Immunohistochemical detection of USP39 expression in lung cancer cells and normal tissues. (d) WB detection of USP39 expression in lung cancer cells and normal tissues. ^∗^*p* < 0.05 versus normal tissues.

The expression levels of miR-381 and USP39 in NSCLC tissues and adjacent normal tissues were detected by qRT-PCR, and the expression level of USP39 was detected by the WB assay. qRT-PCR results showed that the expression of miR-381 in normal human bronchial epithelial cell lines (HBE) was lower than that in A549 cell lines (Figure 1(a)). The expression of USP39 mRNA in lung cancer tissues detected by qRT-PCR was higher than that in the normal control group (Figure 1(b)). This indicates that the expression of miR-381 is correlated with lung cancer cells, and miR-381 may participate in the occurrence and development of non-small-cell lung cancer and inhibit the proliferation of lung cancer cells.

Next, we examined the expression of USP39 in lung cancer cells. The immunohistochemical assay (IHC) was used to detect the expression of USP39. The experimental results show that USP39 expression was significantly upregulated in A549 cell lines compared to HBE cell lines (Figure 1(c)). WB experiments further demonstrated that USP39 was upregulated in A549 cells (Figure 1(d)). In summary, these results suggest that USP39 is highly expressed in NSCLC and may promote the development of lung cancer cells and suggested that USP39 may be a new therapeutic target for NSCLC.

### 3.2. Effects of Overexpression of miR-381 on Proliferation and Invasion of NSCLC Cells

**Figure 2:**
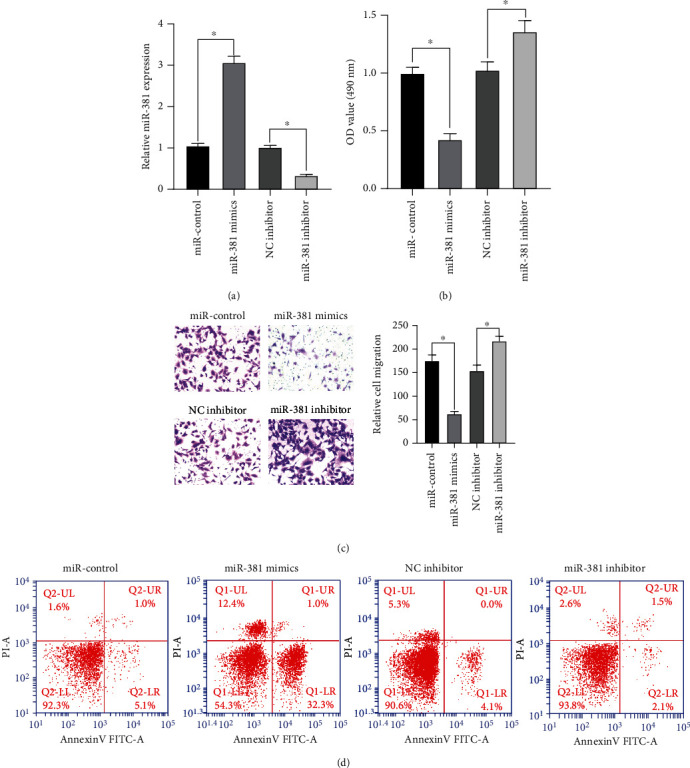
Effect of miR-381 expression on proliferation and invasion of A549 cells. (a) The expression of miR-381 after transfection was detected by qRT-PCR. (b) MTT was used to detect the proliferation activity of cells. (c) Transwell invasion assay was used to analyze the effect of miR-381 on cell invasion. (d) Flow cytometry was used to detect the number of cell apoptosis, and the effect of miR-381 on cell apoptosis was analyzed. ^∗^*p* < 0.05 versus the miR-con group and the NC inhibitors group.

We continued to study the effect of miR-381 expression in NSCLC cells on the invasion and migration of NSCLC cells and transfected miR-381 mimics and miR-381 inhibitors in A549 cells. The expression of miR-381 after transfection was detected by qRT-PCR, and the results showed that miR-381 expression was significantly upregulated in the cells transfected with miR-381 mimics. The expression of miR-381 was significantly reduced in the cells transfected with miR-381 inhibitors (Figure 2(a)). The MTT assay showed that cell proliferation was decreased after transfection with miR-381 mimics. Inhibition of miR-381 expression resulted in increased cell proliferation (Figure 2(b)). On the other hand, the invasion ability of transfected A549 cells was detected by the Transwell invasion assay. The invasion ability of the cells transfected with miR-381 mimics was significantly decreased, while the invasion ability of the cells transfected with miR-381 inhibitors was observably increased (Figure 2(c)). Flow cytometry showed that the apoptosis rate of cells transfected with miR-381 mimics was significantly increased, while the apoptosis rate of cells transfected with miR-381 inhibitors was decreased (Figure 2(d)). Therefore, it can be concluded from the experimental results that the expression of miR-381 can regulate the development of lung cancer cells, and overexpression can increase the apoptosis rate of lung cancer cells.

### 3.3. miR-381 Regulates the Development of Tumor Cells by Targeting USP39

**Figure 3:**
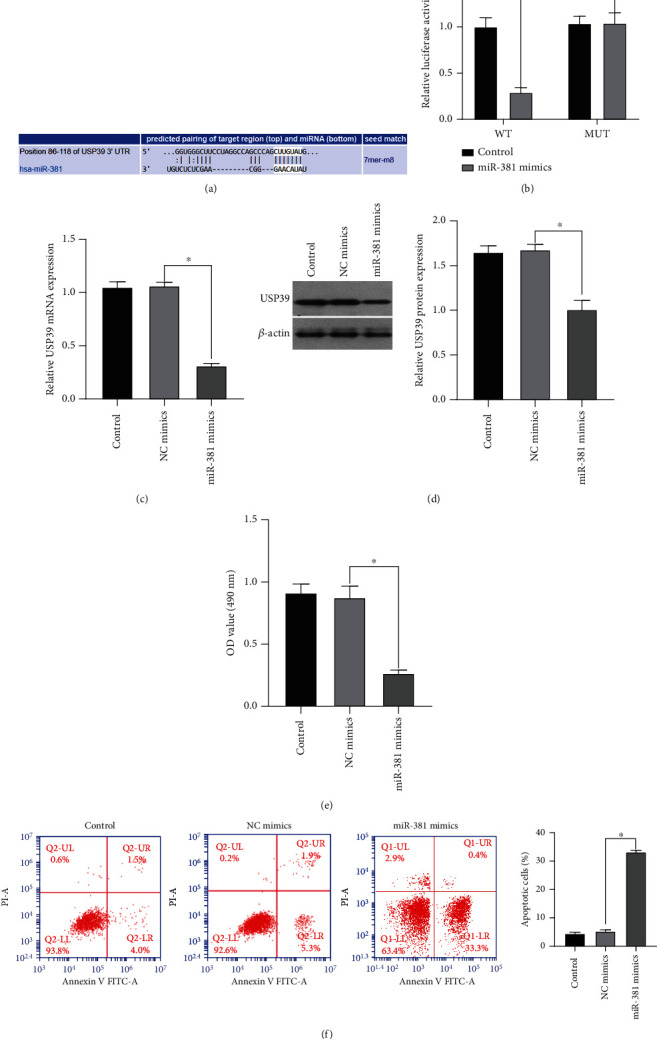
miR-381 regulates USP39 expression in NSCLC cells. (a) TargetScan predicted the binding sequence of miR-381 and USP39. (b) Dual-luciferase analysis of the binding between miR-381 and USP39 3′ UTR sequences. (c) miR-381 mimics were transfected into A549 cells, and the expression of USP39 in cells was detected by qRT-PCR. (d) WB assay was used to analyze the effect of miR-381 overexpression on USP39 expression. (e) The effect of overexpression of miR-381 on A549 cell viability was analyzed by MTT assay. (f) Flow cytometry was used to analyze the effect of miR-381 regulating the expression of USP39 on apoptosis. ^∗^*p* < 0.05 versus the control and NC mimics.

The target prediction of miR-381 and USP39 was analyzed using bioinformatics software TargetScan. The analysis results showed that miR-381 has a targeted binding site with USP39 3′-UTR (Figure 3(a)). HEK-293T cells were selected as the transfected cells in the double luciferase reporter assay because HEK-293T cells had high transfection efficiency and reduced individual differences caused by transfection efficiency. The posttranscriptional luciferase activity was detected by the double luciferase assay. Only miR-381 and USP39-WT 3′UTR cotransfection group showed decreased enzyme activity after simultaneous transfection of all four groups (Figure 3(b)). Experimental results showed that USP39 had binding sites with miR-381. Next, we used qRT-PCR and WB method to detect whether the expression of miR-381 and the expression of USP39 in cells were influenced by each other. The expression of USP39 was significantly reduced in cells transfected with miR-381 mimics (Figures 3(c) and 3(d)). The MTT assay showed that cell viability was decreased after transfection with miR-381 mimics (Figure 3(e)). The number of cell apoptosis was detected by flow cytometry, and the number of cell apoptosis in the transfected miR-381 mimics group was significantly increased, which was higher than that in the normal control group (Figure 3(f)). Experimental data showed that USP39 was a direct target of miR-381, and miR-381 could negatively regulate the expression of USP39 to control tumor proliferation and invasion.

### 3.4. The Overexpression of USP39 Can Reduce the Inhibitory Effect of miR-381

**Figure 4:**
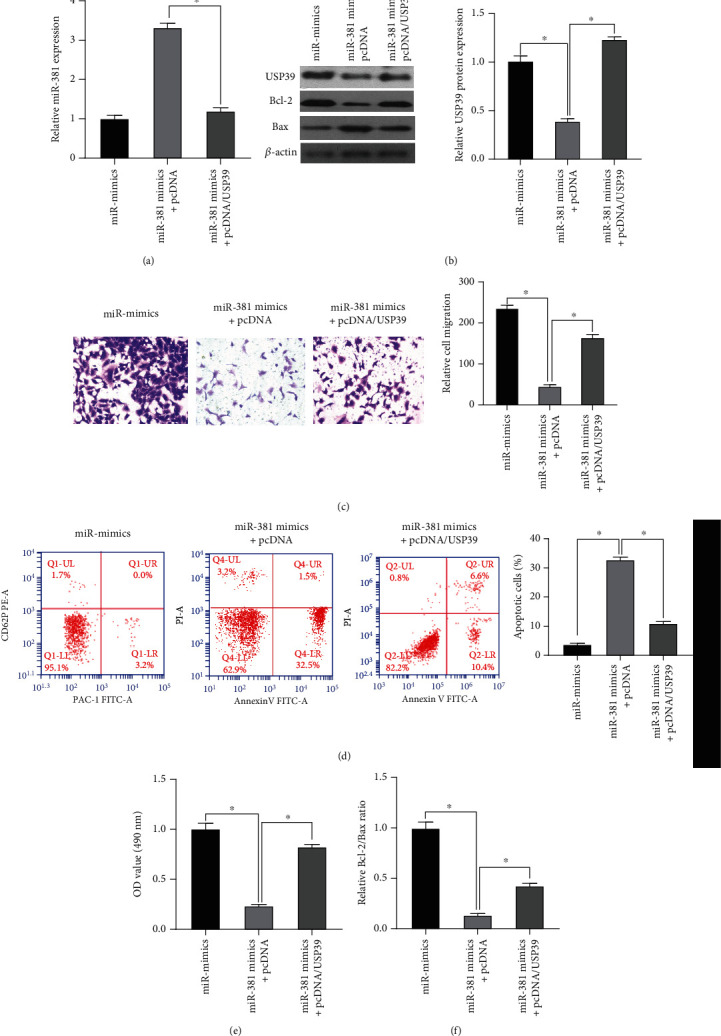
The effect of USP39 overexpression on miR-381 expression. (a) The expression of miR-381 was detected by qRT-PCR after cotransfection of miR-381 and pcDNA/USP39. ^∗^*p* < 0.05 versus the control. (b) The expression of USP39, Bcl-2, and Bax proteins in miR-381 or pcDNA/USP39 cotransfected A549 cells was detected by western blotting. (c) Transwell assay showed the invasion ability of A549 cells cotransfected with miR-381 and pcDNA/USP39. (d) Flow cytometry was used to detect the number of apoptotic cells and to show the effect of cotransfection on the apoptosis of A549 cells. (e) Cell viability of cotransfected cells was detected by MTT. (f) The Bcl-2/Bax ratio showed a decrease in cell viability. ^∗^*p* < 0.05 versus the control and miR-381 mimics.

To verify the effect of USP39 expression on the proliferation and development of NSCLC cells, we cotransfected A549 cells with miR-381 mimics and USP39 plasmid vector. The results of qRT-PCR showed that the expression of miR-381 was inhibited and decreased after cotransfection of miR-381 mimics with the pcDNA/USP39 vector (Figure 4(a)). The WB assay showed that the expression of USP39 was decreased in miR-381 mimics transfected cells, and the result was significantly downregulated compared with the control group (Figure 4(b)).

The Transwell invasion assay showed that expression of USP39 reversed the inhibition of miR-381 mimics, and the invasion ability of cells in the cotransfected pcDNA/USP39 group increased significantly (Figure 4(c)). The number of apoptosis was detected by flow cytometry. Compared with other groups, the number of apoptosis of cells transfected with both miR-381 mimics and USP39 was significantly reduced (Figure 4(d)). The MTT assay was used to detect the viability of transfected cells. Compared with the control group, the viability of transfected cells in the miR-381 mimics group was decreased, and the viability of cells cotransfected with pcDNA/USP39 was increased (Figure 4(e)). The expression of Bcl-2 and Bax proteins in transfected cells was detected by western blot, and the apoptosis status of cells was further detected. After transfection with the miR-381 simulation group, the expression of Bax protein was upregulated, and the expression of Bcl-2 was inhibited (Figure 4(a)). The ratio of Bcl-2/Bax protein in different cells was calculated. miR-381 mimics decreased the ratio of Bcl-2/Bax protein after pcDNA/USP39 cotransfection, while upregulating the ratio of Bcl-2/Bax protein (Figure 4(f)). The results showed that the inhibition of miR-381 could be reversed by the expression of USP39, further confirming that USP39 is the target gene of miR-381.

## 4. Discussion

Nowadays, with the aggravation of environmental pollution, the increase of social pressure, and the bad habits in life, people's life and health are facing more and more threats, and the incidence of cancer is also increasing. Lung cancer is becoming a serious and common cancer. A large number of survey results show that the incidence of lung cancer worldwide is extremely high; the death rate is also in the forefront of the world. Therefore, it is very necessary and meaningful to explore the mechanism of lung cancer.

MicroRNAs (miRNAs) have been widely accepted as an important regulatory factor of human cancer and are involved in the pathogenesis of most cancers. In recent years, more and more studies have been conducted on miRNA in lung cancer. Zhang [19] found that miR-143 can inhibit the proliferation and invasion of NSCLC cells by inhibiting the action of EGFR. The Huang et al. [20] study shows that miR-140-3p in lung squamous cell carcinoma could induce the growth and development of tumor cells and improve the prognosis of patients by regulating the expression of BRD9. Studies have shown [21-23] that miR-381 can exert effects on human cancers such as ovarian cancer, oral squamous cell cancer, and renal cancer by regulating specific genes, but there are few studies on lung cancer [21–23]. Huang et al. found that in NSCLC, expression of miR-381 can slow down cell proliferation and reduce cell carcinogenicity [24]. Multiple studies have shown that miR-381 regulates tumor development by inhibiting a variety of genes in a variety of tumors. In this paper, through bioinformatics analysis, we selected USP39 as the target gene. In this study, we found that the expression of miR-381 was significantly downregulated in non-small-cell lung cancer tissues and cells. Experiments showed that miR-381 could participate in the growth cycle of NSCLC cells, regulate cell proliferation, and increase the mortality of NSCLC cells, which indicated that miR-381 had an inhibitory effect on tumor development in lung cancer cells.

USP39 is a member of the ubiquitin-specific protease family, which is involved in regulating cell mitosis and controlling cell growth and reproduction [5]. USP39 has been found to be associated with the occurrence, progression, and poor prognosis of a variety of cancers, including melanoma, osteosarcoma, and renal carcinoma [25–27]. Recently, studies have shown that USP39 is differentially expressed in lung cancer cells and is related to the development and progression of lung cancer cells and can promote the occurrence and development of tumor cells. For example, Yuan et al. [13] found that USP39 is upregulated in NSCLC tissues, and USP39 knockout can inhibit the growth and metastasis of NSCLC cells. However, the mechanism by which USP39 participates in NSCLC cell proliferation needs further research. In this study, we found that the expression of miR-381 and USP39 was negatively correlated. USP39 was a direct target of miR-381, and overexpression of miR-381 could target and regulate the expression of USP39. In this study, miR-381 mimics could downregulate the expression of USP39 and eventually induce the decrease of the Bcl-2/Bax ratio, control the growth and development of lung cancer cells, and promote the apoptosis of lung cancer cells. However, overexpression of USP39 could reverse this trend, indirectly indicating that miR-381 and USP39 had direct binding sites. These results demonstrate that miR-381 can induce the proliferation and invasion of NSCLC tissues by targeting USP39.

## 5. Conclusion

In conclusion, this study showed that miR-381 was upregulated in NSCLC, and miR-381 effectively controls the proliferation of NSCLC cells by targeting the expression of USP39. These results suggest that USP39 is a valuable therapeutic target for lung cancer. However, the regulatory mechanism of miR-381 and USP39 in NSCLC remains unclear for the time being, and further studies are needed to confirm it.

## Data Availability

The data used to support the findings of this study are available from the corresponding author upon request.
